# Impact of the Affordable Care Act on participation in the Supplemental Nutrition Assistance Program among low-income older Medicare beneficiaries

**DOI:** 10.1186/s12913-023-09557-7

**Published:** 2023-05-19

**Authors:** Hyunmin Kim, Asos Mahmood, Cyril F. Chang, Noah E. Hammarlund, Aram Dobalian

**Affiliations:** 1 School of Health Professions, The University of Southern Mississippi, 118 College Drive #5122, Hattiesburg, MS 39406 USA; 2 Center for Health System Improvement, College of Medicine, University of Tennessee Health Science Center, Memphis, TN USA; 3 Department of Medicine-General Internal Medicine, College of Medicine, University of Tennessee Health Science Center, Memphis, TN USA; 4 Department of Economics, Fogelman College of Business and Economics, The University of Memphis, Memphis, TN USA; 5 Department of Health Services Research, Management and Policy, University of Florida, Gainesville, FL USA; 6 Division of Health Services Management and Policy, College of Public Health, The Ohio State University, Columbus, OH USA

**Keywords:** Supplemental Nutrition Assistance Program, Health Care Reform, Affordable Care Act, Medicare and Medicaid, Dually eligible beneficiaries, Low-income, Older adults

## Abstract

**Background:**

The Affordable Care Act (ACA) provisions, especially Medicaid expansion, are believed to have “spillover effects,” such as boosting participation in the Supplemental Nutrition Assistance Program (SNAP) among eligible individuals in the United States (US). However, little empirical evidence exists about the impact of the ACA, with its focus on the dual eligible population, on SNAP participation. The current study investigates whether the ACA, under an explicit policy aim of enhancing the interface between Medicare and Medicaid, has improved participation in the SNAP among low-income older Medicare beneficiaries.

**Methods:**

We extracted 2009 through 2018 data from the US Medical Expenditure Panel Survey (MEPS) for low-income (≤ %138 Federal Poverty Level [FPL]) older Medicare beneficiaries (*n* = 50,466; aged ≥ 65), and low-income (≤ %138 FPL) younger adults (aged 20 to < 65 years, *n* = 190,443). MEPS respondents of > %138 FPL incomes, younger Medicare and Medicaid beneficiaries, and older adults without Medicare were excluded from this study. Using a quasi-experimental comparative interrupted time-series design, we examined (1) whether ACA’s support for the Medicare-Medicaid dual-eligible program, through facilitating the online Medicaid application process, was associated with an increase in SNAP uptake among low-income older Medicare beneficiaries, and (2) in the instance of an association, to assess the magnitude of SNAP uptake that can be explicitly attributed to the policy’s implementation. The outcome, SNAP participation, was measured annually from 2009 through 2018. The year 2014 was set as the intervention point when the Medicare-Medicaid Coordination Office started facilitating Medicaid applications online for eligible Medicare beneficiaries.

**Results:**

Overall, the change in the probability of SNAP enrollment from the pre- to post-intervention period was 17.4 percentage points higher among low-income older Medicare enrollees, compared to similarly low-income, SNAP-eligible, younger adults (*β* = 0.174, *P* < .001). This boost in SNAP uptake was significant and more apparent among older White (*β* = 0.137, *P* = .049), Asians (*β* = 0.408, *P* = .047), and all non-Hispanic adults (*β* = 0.030, *P* < .001).

**Conclusions:**

The ACA had a positive, measurable effect on SNAP participation among older Medicare beneficiaries. Policymakers should consider additional approaches that link enrollment to multiple programs to increase SNAP participation. Further, there may be a need for additional, targeted efforts to address structural barriers to uptake among African Americans and Hispanics.

**Supplementary Information:**

The online version contains supplementary material available at 10.1186/s12913-023-09557-7.

## Background

The Supplemental Nutrition Assistance Program (SNAP) is a federally funded and state-supervised nutrition program. It has played a critical role in reducing food insecurity among low-income households in the United States (US) by providing a monthly cash benefit for purchasing eligible food items [1]. As the nation’s largest anti-hunger program, an estimated 40 million Americans received SNAP benefits among the 47 million people who were eligible in 2016 [2]. SNAP is considered to be one of the most effective federal anti-poverty programs in reducing poverty and in alleviating food insecurity for millions of low-income Americans [3]. To date, however, SNAP participation among a particularly needy population, i.e. eligible low-income seniors, remains low. In 2017, for example, 82% of the general population eligible for SNAP participated in this program, while only 42% of eligible older adults did [4].

The Affordable Care Act (ACA) implementation is believed to have direct or spillover implications for reducing food insecurity and boosting SNAP participation in the US, particularly, through the expansion of health insurance coverage [5, 6]. Generally, the ACA aimed to achieve the triple aims of better healthcare, better health outcomes, and better value by providing increased access to healthcare for the US population [7]. It included several measures targeted at older Medicare populations with a focus on those who were eligible for both Medicare and Medicaid, the so-called “dual eligibles.” It specifically included a set of options to provide enhanced care to dual eligibles such as improved care coordination, increased access to long-term care services, and enhanced performance measures to monitor progress. To achieve these aims, the ACA constructed two entities: The Center for Medicare and Medicaid Innovation (CMMI) and the Medicare-Medicaid Coordination Office (MMCO) or “Duals Office.” The Duals Office has several programmatic goals[8] including offering dually eligible individuals full access to Medicare and Medicaid benefits and simplifying associated processes.

Dual eligibles are defined as Medicare enrollees who are also eligible for Medicaid either due to their income status or because of costly medical bills upon which they can have full or partial dual benefits. It is estimated that about 12 million people, or about 19% of Medicare and 14% of Medicaid enrollees are dually eligible [9]. Dual eligibles are considered the most vulnerable patient population in the US public health system [10] as they are some of the most chronically ill persons and require a higher amount of spending in both programs [11–14]. To target the dual-eligible population for assistance, the ACA made several changes including standardizing the eligibility criteria and benefits for Medicaid and designating the Duals Office as the lead agency to coordinate benefits for this population. Importantly, the ACA simplified the application process by creating an online application system [14].

Although Medicare provides near-universal insurance coverage, many low-income Medicare beneficiaries experience financial challenges related to paying medical bills [15, 16]. Some of these challenges are likely due to the tradeoff between paying for essential food and medical care that confronts some Medicare beneficiaries [15, 16]. Relatedly, older adults living at or below the poverty threshold were found to be more likely to visit emergency departments (ED) and be hospitalized compared to their counterparts with higher incomes [17]. SNAP could help eligible low-income older adults with higher health needs be able to access needed medical care without having to forgo food and proper nutrition, which ultimately could lead to reduced hospitalizations and ED visits, as well as decreased healthcare costs [17]. Nevertheless, many who are eligible to receive SNAP and would benefit from it do not enroll.

Numerous studies provide evidence of the benefits of SNAP enrollment. For example, increased access to a nutritional diet through SNAP could help improve nutrition-sensitive chronic conditions (e.g., hypertension, diabetes, obesity, etc.) in the long run [18]. Additionally, SNAP benefits can reduce adverse health behaviors, including medication non-adherence [19] and non-discretionary healthcare use, such as ED visits and hospital admissions [20]. For example, in a cohort study, Berkowitz and colleagues investigated the relationship between SNAP enrollment and health care use and costs among dually eligible older adults (aged ≥ 65 years) in North Carolina and found that SNAP participation was positively related to lower ED use and fewer in-hospital and long-term care admissions, as well as a $2,360 less yearly Medicaid spending per person [21]. Similarly, Samuel et al. examined the question of whether SNAP participation was associated with lower subsequent ED and hospital utilization among low-income older adults living in Maryland and found that dual eligibles participating in SNAP had a 10% and a 14% lower chance of using both services relative to their counterpart non-SNAP participants, respectively [17]. Further, SNAP participation has been attributed to reduced poverty, improved mental health and psychological well-being, improved overall quality of life, and better health outcomes [22–24].

Only a handful of studies have thus far examined the impact of the ACA on social safety net programs, including SNAP. For instance, Burney and colleagues assessed the role of the ACA on SNAP participation and found that the Medicaid expansion was related to a 3.18% increase in the chance of enrolling in SNAP in a household [9]. Furthermore, Schmidt et al. found that Medicaid expansions positively affected SNAP participation among low-income individuals, particularly in regions where the SNAP uptake rate was initially low [25]. Nonetheless, those studies did not focus on the low-income older Medicare population. The purpose of this study is to examine whether the ACA’s efforts to facilitate the Medicaid application process for those who are dual-eligible were associated with increased participation in SNAP among low-income older Medicare enrollees. Given the comparatively low SNAP participation among eligible seniors and the program’s beneficial effects in improving health, quality of life, and health outcomes [17–19, 21, 23, 24, 26, 27], it is worthwhile to further explore this question. We hypothesize that the ACA, under an explicit policy aim of enhancing the interface between Medicare and Medicaid, boosted SNAP participation among low-income Medicare beneficiaries.

## Methods

### The conceptual framework

**Fig. 1:**
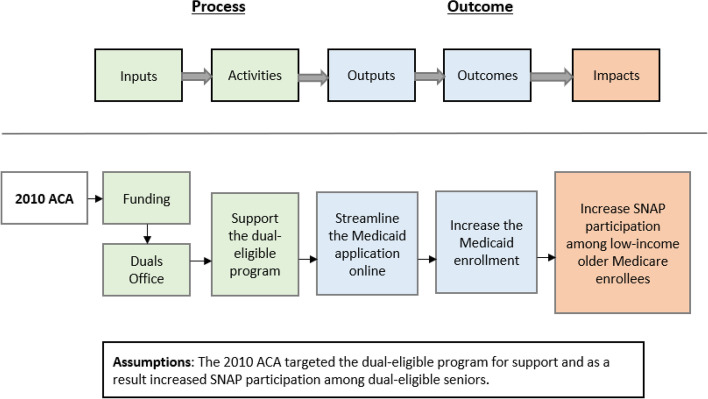
A logic model for evaluating the impact of the Affordable Care Act (ACA) on Supplemental Nutrition Assistance Program (SNAP) participation among low-income older Medicare beneficiaries in the US

This study was conceptually based on a program planning tool called the Program Logic Model to measure and assess the potential impact of the ACA on SNAP participation [28, 29]. Adhering closely to the Logic Model’s emphasis on inputs, outputs, and outcomes, we developed a conceptual framework of program evaluation as shown in Fig. 1 to guide the design and development of our evaluation model.

Our Logic Model begins with two assumptions: (1) the 2010 ACA supports the dual eligible program; and (2) an increase in SNAP participation among low-income older Medicare enrollees can be attributed to the ACA’s 2014 feature of facilitating the Medicaid application process for dual eligibles. Specifically, the section 2602 (c) of the ACA mandated the establishment and operation of the Duals Office to ensure that dual eligibles can access benefits to both programs more effectively and enhance coordination or cooperation between the Federal and State governments concerning offering those benefits. Further, beginning in 2014, the ACA offered harmonized, streamlined application processes for the “insurance affordability programs”, including Medicaid [30]. The final rule to enforce the Medicaid-related provisions, including simplifying the application process, was effective since January 1, 2014. The application system and its associated assistance (including providing information on eligibility criteria, services that are covered, etc.) must also be provided online in addition to by mail, by telephone, and in-person. One main factor that could contribute to increased SNAP uptake among low-income older Medicare beneficiaries is the ease with which Medicaid-enrollee data can be connected with SNAP-enrollee files to assist in detecting dual-eligible older persons who are eligible for but have not yet enrolled in SNAP [31]. Furthermore, state Medicaid programs serve as an excellent entry point for beneficiaries to access a variety of additional social services [31]. This is in addition to the fact that raising awareness of social programs and giving more information would also likely impact uptake [32]. Low-income older Medicare beneficiaries may otherwise be unaware of their eligibility and benefits under the SNAP program, and thus participation in Medicaid could provide an opportunity to obtain the entire range of SNAP program benefits. Therefore, our model posits that the ACA’s mechanism streamlining the application process available online contributed to an increase in Medicaid enrollment with a spillover effect on increasing SNAP participation among low-income older Medicaid enrollees.

In this Logic Model, ‘inputs’ pertain to the financial and human resources required to implement a program or policy and correspond to funding provided to the Duals Office attributed to the 2010 ACA. ‘Activities,’ defined as processes of converting inputs to outputs, can be seen as actions taken by the Dual Office in support of the dual eligible program. ‘Outputs’ refers to the product/service that is completed/delivered and corresponds to streamlining the Medicaid application process online. ‘Outcomes’ indicates the intended or anticipated changes attributed to a policy or intervention and here represents an increase in Medicaid enrollment among dual eligibles. Finally, ‘Impacts’ refers to changes in the outcome, and corresponds to increased SNAP participation among low-income older Medicare enrollees.

### Data sources, analyzed sample, and the study cohorts

This study employed the 2009 through 2018 cycles of data from the IPUMS Medical Expenditure Panel Survey (MEPS), sponsored by the Agency for Healthcare Research and Quality and co-sponsored by the Centers for Disease Control and Prevention’s National Center for Health Statistics. The IPUMS Health Surveys: MEPS dataset, is an integrated, harmonized, multiyear version of the publicly available files of US MEPS, which is a nationally representative survey of non-institutionalized individuals and their families, care providers and employers [33, 34]. More details concerning the survey design, methodology, and access to IPUMS MEPS data can be found online @ https://meps.ipums.org/meps/userGuide.shtml.

To adequately capture the true ACA impact on low-income Medicare enrollees’ SNAP participation, it is insufficient to do a simple “before-after” comparison of the study sample because of the potential biases that can be introduced by the many other factors that could have also influenced the complicated and evolving participation decisions over time. To isolate the study effect, we followed the spirit of the “difference-in-differences” empirical design and selected first a study group by extracting longitudinal data from the database for all low-income older (ages 65 +) Medicare enrollees who were eligible for SNAP benefits (*n* = 50,466). For comparison, a cohort of younger (aged 20 to < 65 years) low-income individuals was selected as the control group (*n* = 190,443) who met the same set of SNAP eligibility requirements.

For both groups, low-income status was determined based on the Census Bureau’s income threshold of ≤ 138% of the Federal Poverty Levels (FPL), which overlaps with SNAP eligibility in general, albeit with some differences across the States. We chose this threshold, in part, to conform to the ACA’s use of 138% FPL as the effective minimum income floor for expanded Medicaid eligibility. Thus, we excluded all other individuals with incomes > 138% of the FPL. We also excluded younger individuals with dual Medicare and Medicaid coverage as well as older adults without Medicare. Further, low-income adults aged 18–20 years were not included in the control group because they were potentially covered by the Children’s Health Insurance Program (CHIP). We recognize that the two heterogeneous age groups can reasonably be assumed to differ in how they see the SNAP program, as well as in how they respond to participation incentives. However, the application of a two-group design to a nationally representative database spanning over a sufficiently long period both before and after the implementation of ACA should minimize potential biases. Moreover, the longitudinal difference in the between-group difference in SNAP participation is our focus in this study.

A series of sociodemographic characteristics for the two study cohorts were analyzed and reported. These included survey respondents’ self-reported age, sex, marital status, race, educational attainment, income, and comorbid medical conditions (including diabetes, cancer, asthma, and coronary heart disease). The outcome of interest, “SNAP participation,” was measured annually over the investigated period (i.e., 2009 – 2018). Since we employed de-identified and publicly available data; our study did not require additional institutional review board approval as it did not qualify as human subjects research [35]. All results were reported in accordance with the Reporting of studies Conducted using Observational Routinely-collected health Data (RECORD) statement [36].

### The empirical framework and estimation approach

We used a comparative interrupted time series (ITS) method to empirically assess the potential impact of the ACA on SNAP participation among low-income older Medicare enrollees. Generally considered a strong quasi-experimental design for assessing the effects of a policy or interventions, the ITS method has been utilized to examine the effect of an interruption (policy change) on a specific outcome with multiple measurements. The ITS approach is based on an outcome with multiple measurements assessed at different points in time before and after an interruption or intervention. Specifically, our outcome variable was measured in each year between 2009 and 2018 and includes the year 2014 when the Duals Office began facilitating online Medicaid applications.

In this instance, ITS models can assess policy outcomes in greater detail by separating “level” or discrete changes in the probability of SNAP enrollment from those measured at the margin or reflected by the “slope” of the relationship between the pre- and post-interruption periods. This aspect of ITS models is particularly suited for policy evaluation when the study data allow for both a control versus treatment comparison and a pre- and post-comparison. For example, temporal trends (i.e., the level and trajectory of SNAP participation) in the control group can be captured by subtracting the pre/post change in the control group from that in the intervention group [37]. Similarly, the effects of time-varying confounders that potentially affect evaluation outcomes, including other events that took place at the same period, can be controlled and measured. In 2014 (i.e., the current study’s intervention year) for instance, there were several key features of the ACA that could have similar influences on the outcomes, including tax credits for purchasing insurance and Medicaid expansion. The main assumption in the model is that the change in the level or trend in SNAP participation as the outcome of interest is assumed to be the same both for the low-income younger group and the older Medicare group with low incomes as they have not been exposed to the policy change or intervention as counterfactual. That is, it is presumed that all confounding factors or covariates that were omitted may influence both groups similarly.

In this analysis, the treated group comprised low-income older Medicare enrollees who were eligible for SNAP benefits. This group was compared to younger low-income individuals who met the same SNAP eligibility requirements. Consider a comparative ITS model in the following form:

$$Y_t=\beta_0+\beta_1T_t+\beta_2X_t+\beta_3T_t\cdot X_t+\beta_4Z+\beta_5Z\cdot T_t+\beta_6Z\cdot X_t+\beta_7Z\cdot X_t\cdot T_t+\varepsilon_{t,}$$

where Y_t_ is a binary outcome variable, with 1 indicating SNAP participation at time t, and 0 otherwise; and subscript t denotes years 2009 through 2018. On the right-hand side of the equation, the independent variable ‘T_t_’ shows the trend (or slope) in the probability of SNAP enrollment in the control group before interruption/intervention, recoded as a continuous variable (‘1’ for the year 2009 to ‘10’ for year 2018). The independent variable ‘X_t_’ indicates interruption or intervention, recoded as a dummy variable taking on the value of 0 for the pre-interruption/intervention period of 2014, before the establishment of the Dual Office, and 1 for the post-interruption/intervention period. The interruption or intervention was defined as occurring in the year 2014 when the Duals Office started facilitating the Medicaid application online for eligible Medicare enrollees. The independent variable ‘X_t_∙T_t_’ represents the interaction between X_t_ and T_t_ and indicates the time after interruption/intervention, recoded as a continuous variable (‘0’ for years 2009 to 2013, ‘1’ for the year 2014, to ‘5’ for year 2018).

A third main independent variable of interest, ‘Z,’ shows treatment/exposure and is coded as binary (‘1’ for low-income older Medicare enrollees and ‘0’ for low-income younger individuals). The independent variable ‘Z∙T_t_’ as an interaction of Z and T_t_ indicates the time for treatment, recoded as a continuous variable. The independent variable ‘Z∙X_t_’ as an interaction of Z and X_t_ shows the interruption for the treatment group, recoded as a continuous variable. The independent variable ‘Z∙X_t_ ∙T_t_’ as an interaction of Z, X_t_, and T_t_ indicates the time after interruption/intervention. The coefficient β_7_, as a main interest of the study, shows the difference between the low-income Medicare group and the low-income younger adult group in the slope (or trend) and represents the probability of SNAP participation after the interruption/intervention compared to pre-interruption/intervention. Finally, Ɛ_t_ is an error term that can capture unobserved factors and any possible measurement errors.

### Statistical analysis

We first analyzed and reported means and proportions for study participant characteristics. ITS analysis was then conducted with robust standard errors using the generalized estimating equation, which generates efficient coefficient estimates. Further, we tested for autocorrelation (or serial correlation) using the Ljung-Box Test [38] to see whether SNAP participation assessed at some time may be correlated with its past measured values. From the result, we detected the presence of autocorrelation (*P* < 0.001); thus, we used the Newey-West autocorrelation adjusted standard errors. Additionally, we performed subgroup analysis by race and ethnicity to see if there existed racial/ethnic heterogeneity in the policy impact on SNAP participation.

To check for robustness and validity, we conducted several additional analyses, including sensitivity analyses. We first checked whether the results would be different by excluding individuals aged 60 to 64 years. The rationale behind this exclusion is that those aged 60 years or older are subjected to special rules for SNAP eligibility [39]. Thus, by excluding those aged 60 to 64 years, we could have addressed the possibility that special rules that apply to non-Medicare older adults may have an influence. We also performed analyses by excluding individuals aged 20 to 26 years in our control group, given that some of those individuals could be attached to their parents’ health insurance plan. Furthermore, to address the potentiality of differences in characteristics between the treatment and control groups that may affect our results, we further conducted ITS analyses by incorporating a propensity score matching. Specifically, we performed ITS analyses using the data that matched each older Medicare enrollee to the younger based on propensity score, calculated based on the covariates (age, sex, race, education, marital status, and income). Additionally, we conducted a sensitivity analysis reflecting a lower poverty threshold (≤ 130% of the FPL), given that not all states have the same income eligibility requirement for Medicaid, recognizing that the current study involves the intervention of the Dual Office, not the ACA Medicaid expansion.

All statistical analyses were performed using the SAS software program version 9.4 and the R program version 4.2.3. The significance assessment was carried out at the threshold level of *P* < 0.05.

## Results

Table 1 presents descriptive characteristics of older Medicare enrollees and their low-income younger counterparts. Overall, SNAP participation rates were higher in the low-income younger group than in the low-income older Medicare group (23.4% versus 15.2%). The mean age of the low-income older Medicare enrollees was 74.7 years (versus 37.7 years for the low-income younger). Most individuals in both groups were White (78.3%, 73.5%), women (62.8%, 61.3%), and GED/high school graduates (32.6%, 37.8%). The older Medicare individuals were more likely to be married (51.8%) while the younger group was mainly never-married (44.2%). The mean annual income was higher among older Medicare enrollees relative to the younger individuals ($8,827.9 versus $6,223.7). Diabetes, cancer, and coronary heart disease were more prevalent among the older Medicare group than the younger low-income group.

**Table 1:** Characteristics of low-income older Medicare enrollees and the low-income younger population (The United States, years 2009 through 2018, Medical Expenditure Panel Survey)

	**Older low-income** **Medicare enrollees** **(aged ≥ 65, *n* = 50,466)**		**Younger low-income adults (aged 20 to < 65 years, *n* = 190,443)**	
Characteristics	% or Mean	SE or SD	% or Mean	SE or SD
SNAP participation	15.2	0.58	23.4	0.63
Age (in years)	74.7	6.71	37.7	13.39
Sex				
Male	37.2	0.49	38.7	0.34
Female	62.8	0.49	61.3	0.34
Marital status				
Married	51.8	0.9	43.2	0.51
Divorced/Separated/Widowed	43.1	0.83	12.6	0.25
Never married	5.1	0.28	44.2	0.45
Race				
White	78.3	0.97	73.5	0.93
Black	13.2	0.7	15.9	0.75
Asian	6.1	0.59	6.5	0.37
Other	2.4	0.32	4.1	0.23
Education				
No degree	19.5	0.59	12.1	0.33
GED/High school graduate	32.6	0.65	37.8	0.41
College degree or above	9.7	0.41	10.2	0.29
Not reported	38.2	0.71	39.9	0.39
Income (US$)	8827.9	5068.5	6223.7	5704.4
Comorbidities				
Diabetes	26.4	0.52	7.2	0.16
Cancer	27.5	0.69	5.1	0.15
Asthma	10.7	0.42	11.5	0.27
Coronary heart disease	20.6	0.49	2.4	0.09

Low-income status was determined at ≤ 138% of FPL

*Abbreviations. SNAP* Supplemental Nutrition Assistance Program, *FPL* Federal Poverty Level, *SE* Standard Error, *SD* Standard Deviation

**Fig. 2:**
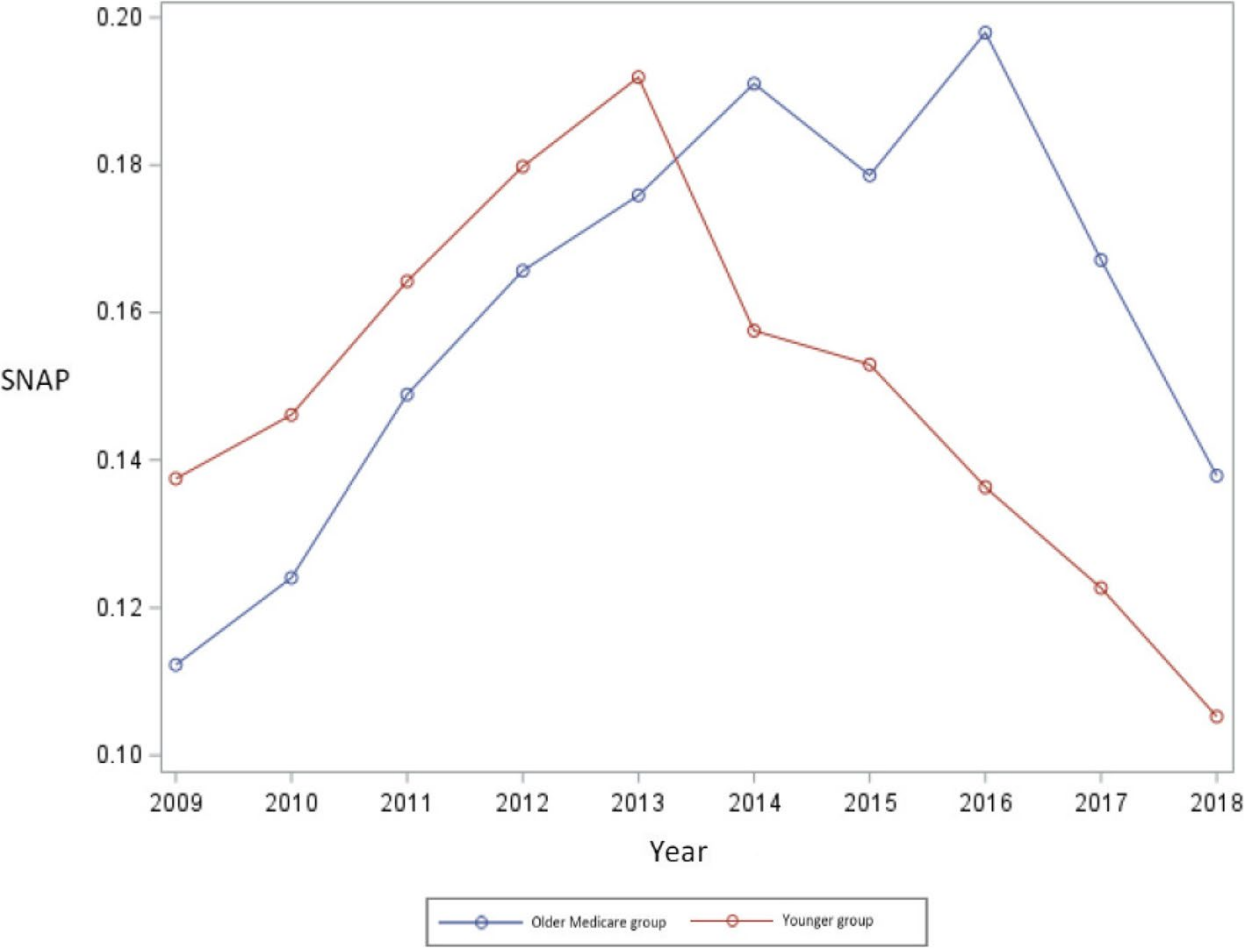
Annual trends of Supplemental Nutrition Assistance Program (SNAP) participation rates among low-income older Medicare enrollees versus the low-income younger adults in 2009 through 2018 in the US

Annual trends of SNAP participation illustrate that before 2014, the rates were heading upward for both groups, with low-income younger adults having higher rates of participation than older Medicare enrollees but with relatively a narrower gap (See Fig. 2). Immediately after 2014, the year the Duals Office began facilitating online Medicaid applications, the trends reversed and older Medicare beneficiaries had higher rates of SNAP participation with a relatively larger difference than low-income younger adults. However, more recently, SNAP participation rates have trended down for both groups equally.

The comparative ITS analysis results are displayed in Table 2. The parameter ‘Time’ represents the trend (or slope) in the likelihood of SNAP participation in the control group before interruption/intervention. This estimate indicates that the probability of SNAP enrollment among the low-income younger group increased by 11 percentage points (PP) in the pre-policy (intervention) period (*β* = 0.11, *P* < 0.001). The parameter ‘Interruption’ shows the segmentation between pre- and post-intervention (i.e. the level change in the outcome) among the control (younger) group. The parameter ‘Time∙Interruption’ shows the trend change (or slope) in the probability of SNAP enrollment after interruption among the control group. This estimate indicates that the trend in the likelihood of SNAP participation among low-income younger adults decreased by 25 PP after the interruption/intervention (*β* =  − 0.252, *P* < 0.001). The parameter ‘Treatment’ indicates the level difference in the probability of SNAP participation before the intervention between the treatment and control groups. The level difference in the likelihood of SNAP enrollment before the intervention between low-income older Medicare enrollees and younger low-income adults was estimated to be 131 PP (*β* = 1.311, *P* < 0.001). The parameter ‘Treatment∙Time’ shows the trend (or slope) difference in the likelihood of SNAP participation before intervention between the treatment and control groups. Our results indicate that the trend (or slope) difference in the probability of SNAP enrollment before the intervention/interruption between the low-income older Medicare group and the low-income younger was estimated to be one (1) PP, albeit not statistically significant. The parameter ‘Treatment∙Interruption’ depicts the level difference in the likelihood of SNAP participation between the treatment and control groups after intervention. This finding shows that the level difference in the probability of SNAP enrollment after the intervention/interruption between the low-income older Medicare group and the younger with low incomes was estimated to be 3.9 PP, however, not statistically significant.

**Table 2:** Comparative ITS analysis examining the ACA policy impact on SNAP participation among low-income older Medicare enrollees

		**SNAP participation**			
Parameter	Interpretation	β	*P*	SE^a^	t
Time^b^	Control Pre–Trend	0.11	< .001	.001	7.97
Interruption^c^	Control Post–Level Change	− 0.032	.52	.007	− 1.38
Time∙Interruption^d^	Control Post–Trend Change	− 0.252	< .001	.002	− 11.33
Treatment^e^	Treatment/Control Pre–Level Difference	1.311	< .001	.028	7.64
Treatment∙Time^f^	Treatment/Control Pre–Trend Difference	− 0.013	.72	.008	1.23
Treatment∙Interruption^g^	Treatment/Control Post–Level Difference	− 0.039	.78	.032	− 0.14
Treatment∙Interruption∙Time^h^	Treatment/Control Change in Slope Difference Pre– to Post–	0.174	< .001	.011	1.07

Low-income status was determined at ≤ 138% of FPL

*Abbreviations*. *ITS* interrupted time series, *ACA* Affordable Care Act, *SNAP* Supplemental Nutrition Assistance Program, *β* unstandardized beta coefficient, *P p*-value, *SE* standard error, *t* t-statistic, *FPL* Federal Poverty Level

^a^Newey-West autocorrelation adjusted standard error

^b^The coefficient of ‘Time’ indicates the trend (or slope) in the probability of SNAP participation in the low-income younger group before interruption/intervention

^c^The coefficient of ‘Interruption’ shows the level change in the likelihood of SNAP participation right after the interruption/intervention in the low-income younger group

^d^The coefficient of ‘Time∙Interruption’ indicates the trend (or slope) change in the probability of SNAP participation after the interruption in the low-income younger group

^e^The coefficient of ‘Treatment’ shows the level difference in the probability of SNAP enrollment before the intervention between the low-income older Medicare group and the low-income younger group

^f^The coefficient of ‘Treatment∙Time’ indicates the trend (or slope) difference in the probability of SNAP participation before intervention between the low-income older Medicare group and the low-income younger group

^g^The coefficient of ‘Treatment∙Interruption’ shows the level difference in the probability of SNAP enrollment between the low-income older Medicare group and the low-income younger group

^h^The coefficient of ‘Treatment∙Interruption∙Time’ indicates the change in slope difference in the likelihood of SNAP participation between the low-income older Medicare group and the younger group with low incomes

The parameter ‘Treatment∙Interruption∙Time’ indicates the main coefficient of interest — the change from pre- to post-intervention in trend difference in the likelihood of SNAP participation between the older Medicare and the younger low-income cohorts (see Table 2). This finding indicates that the difference in trend in the probability of SNAP participation after the interruption/intervention compared to pre-interruption/intervention between the low-income Medicare group and the younger with low incomes was estimated to be 17.4 PP (*P* < 0.001).

We further conducted subgroup analyses by participants’ race and ethnicity and the results are presented in Tables 3 and 4, respectively. Overall, similar patterns were observed in SNAP participation, with some observed differences. Notably, among Whites, the trend difference in the likelihood of SNAP participation, from pre- to post-policy intervention, between low-income older Medicare enrollees and the low-income younger group was estimated to be 13.7 PP (*β* = 0.137, *P* = 0.049). Similar results were shown for Asians, albeit of a different magnitude (*β* = 0.408, *P* = 0.047). However, significant findings were not shown for low-income Blacks or other racial or minoritized individuals.

**Table 3:** Subgroup analysis exploring racial heterogeneity in the ACA policy impact on SNAP participation among low-income Medicare enrollees

	**SNAP participation**							
	**Black**		**White**		**Asian**		**Other**	
Parameter	β (SE)	*P*	β (SE)	*P*	β (SE)	*P*	β (SE)	*P*
Time^a^	0.102 (.022)	< .001	0.111 (.014)	< .001	0.186 (.072)	.011	0.023 (.064)	.72
Interruption^b^	− 0.111 (.095)	.24	− 0.0001 (.064)	.99	0.093 (.292)	.75	− 0.461 (.263)	.08
Time∙Interruption^c^	− 0.213 (.035)	< .001	− 0.267 (.023)	< .001	− 0.236 (.112)	.04	0.005 (.092)	.96
Treatment^d^	0.939 (.232)	< .001	1.398 (.177)	< .001	2.111 (.535)	< .001	0.342 (1.213)	.78
Treatment∙Time^e^	− 0.017 (.069)	.80	0.008 (.051)	.87	− 0.114 (.146)	.43	0.337 (.312)	.28
Treatment∙Interruption^f^	0.193 (.260)	.46	− 0.070 (.192)	.72	− 0.641 (.523)	.22	0.180 (.747)	.81
Treatment∙Interruption∙Time^g^	0.131 (.096)	.17	0.137 (.070)	.049	0.408 (.205)	.047	− 0.475 (.354)	.18

Robust standard errors are in parentheses. Low-income status was determined at ≤ 138% of FPL

*Abbreviations. ACA* Affordable Care Act, *SNAP* Supplemental Nutrition Assistance Program, *β* unstandardized beta coefficient, *P p*-value, *SE* standard error, *FPL* Federal Poverty Level

^a^The coefficient of ‘Time’ indicates trend (or slope) in the probability of SNAP participation in the low-income younger group before interruption/intervention

^b^The coefficient of ‘Interruption’ shows the level change in the likelihood of SNAP participation right after the interruption/intervention in the low-income younger group

^c^The coefficient of ‘Time∙Interruption’ indicates the trend (or slope) change in the probability of SNAP participation after the interruption in the low-income younger group

^d^The coefficient of ‘Treatment’ shows the level difference in the probability of SNAP enrollment before the intervention between the low-income older Medicare group and the low-income younger group

^e^The coefficient of ‘Treatment∙Time’ indicates the trend (or slope) difference in the probability of SNAP participation before intervention between the low-income older Medicare group and the low-income younger group

^f^The coefficient of ‘Treatment∙Interruption’ shows the level difference in the probability of SNAP enrollment between the low-income older Medicare group and the low-income younger group

^g^The coefficient of ‘Treatment∙Interruption∙Time’ indicates the change in slope difference in the likelihood of SNAP participation between the low-income older Medicare group and the younger with low incomes

**Table 4:** Subgroup analysis exploring Hispanic ethnicity heterogeneity in the ACA policy impact on SNAP participation among low-income Medicare enrollees

	**SNAP participation**			
	**Hispanic**		**Non-Hispanic**	
Parameter	β (SE)^a^	*P*	β (SE)^a^	*P*
Time^b^	0.021 (.004)	< .001	0.032 (.003)	< .001
Interruption^c^	0.006 (.014)	.66	0.014 (.011)	.19
Time∙Interruption^d^	− 0.035 (.006)	< .001	− 0.051 (.004)	< .001
Treatment^e^	− 0.005 (.034)	.86	− 0.115 (.014)	< .001
Treatment∙Time^f^	− 0.003 (.012)	.74	− 0.011 (.005)	.03
Treatment∙Interruption^g^	0.015 (.035)	.65	0.002 (.018)	.88
Treatment∙Interruption∙Time^h^	0.015 (.014)	.29	0.030 (.007)	< .001

Robust standard errors are in parentheses. Low-income status was determined at ≤ 138% of FPL

*Abbreviations*. *ACA* Affordable Care Act, *SNAP* Supplemental Nutrition Assistance Program, *β* unstandardized beta coefficient, *P p*-value, *SE* standard error, *FPL* Federal Poverty Level

^a^Newey-West autocorrelation adjusted standard error

^b^The coefficient of ‘Time’ indicates trend (or slope) in the probability of SNAP participation in the low-income younger group before interruption/intervention

^c^The coefficient of ‘Interruption’ shows the level change in the likelihood of SNAP participation right after the interruption/intervention in the low-income younger group

^d^The coefficient of ‘Time∙Interruption’ indicates the trend (or slope) change in the probability of SNAP participation after the interruption in the low-income younger group

^e^The coefficient of ‘Treatment’ shows the level difference in the probability of SNAP enrollment before the intervention between the low-income older Medicare group and the low-income younger group

^f^The coefficient of ‘Treatment∙Time’ indicates the trend (or slope) difference in the probability of SNAP participation before intervention between the low-income older Medicare group and the low-income younger group

^g^The coefficient of ‘Treatment∙Interruption’ shows the level difference in the probability of SNAP enrollment between the low-income older Medicare group and the low-income younger group

^h^The coefficient of ‘Treatment∙Interruption∙Time’ indicates the change in slope difference in the likelihood of SNAP participation between the low-income older Medicare group and the younger with low incomes

Further, the findings from the ethnical heterogeneity analysis indicate that, among all non-Hispanics, the difference in trend in the probability of SNAP participation after the interruption/intervention compared to pre-interruption/intervention between the low-income Medicare group and the younger with low incomes was estimated to be 3.0 PP (*P* < 0.001) (Table 4). However, significant findings were not shown for low-income Hispanic individuals.

To check robustness (see Table 5), we also performed a comparative ITS analysis excluding individuals aged 60–64 years. Although there were some differences, overall similar results were shown. For instance, the estimate of ‘Time’ shows that the likelihood of SNAP participation among low-income individuals aged 20–59 years increased by 10.6 PP in the pre-intervention period (*β* = 0.106, *P* < 0.001). The estimate of ‘Time∙Interruption’ indicates that the slope in the probability of SNAP enrollment among low-income individuals aged 20–59 years decreased by 24.8 PP after the intervention (*β* =  − 0.248, *P* < 0.001). The estimate of ‘Treatment’ shows the level difference in the probability of SNAP participation before the interruption between older Medicare enrollees and younger low-income individuals aged 20–59 years was found to be 128 PP (*β* = 1.281, *P* < 0.001). The estimate of ‘Treatment∙Interruption∙Time’ indicates that the slope difference in the likelihood of SNAP enrollment from the pre-intervention to post-intervention between low-income older Medicare enrollees and younger individuals aged 20–59 years was found to be 17.3 PP (*P* = 0.001).

**Table 5:** Robustness analysis excluding older adults aged 60–64 years

		**SNAP participation**			
Parameter	Interpretation	β	*P*	SE^a^	t
Time^b^	Control Pre–Trend	0.106	< .001	.001	7.55
Interruption^c^	Control Post–Level Change	-0.026	.62	.007	-1.28
Time∙Interruption^d^	Control Post–Trend Change	-0.248	< .001	.002	-10.99
Treatment^e^	Treatment/Control Pre–Level Difference	1.281	< .001	.028	7.52
Treatment∙Time^f^	Treatment/Control Pre–Trend Difference	-0.008	.81	.008	1.26
Treatment∙Interruption^g^	Treatment/Control Post–Level Difference	-0.053	.70	.032	-0.15
Treatment∙Interruption∙Time^h^	Treatment/Control Change in Slope Difference Pre– to Post–	0.173	.001	.011	1.09

Low-income status was determined at ≤ 138% of FPL

*Abbreviations*. *ITS* interrupted time series, *ACA* Affordable Care Act, *SNAP* Supplemental Nutrition Assistance Program, *β* unstandardized beta coefficient, *P p*-value, *SE* standard error, *t* t-statistic, *FPL* Federal Poverty Level

^a^Newey-West autocorrelation adjusted standard error

^b^The coefficient of ‘Time’ indicates the trend (or slope) in the probability of SNAP participation in the low-income younger group before interruption/intervention

^c^The coefficient of ‘Interruption’ shows the level change in the likelihood of SNAP participation right after the interruption/intervention in the low-income younger group

^d^The coefficient of ‘Time∙Interruption’ indicates the trend (or slope) change in the probability of SNAP participation after the interruption in the low-income younger group

^e^The coefficient of ‘Treatment’ shows the level difference in the probability of SNAP enrollment before the intervention between the low-income older Medicare group and the low-income younger group

^f^The coefficient of ‘Treatment∙Time’ indicates the trend (or slope) difference in the probability of SNAP participation before intervention between the low-income older Medicare group and the low-income younger group

^g^The coefficient of ‘Treatment∙Interruption’ shows the level difference in the probability of SNAP enrollment between the low-income older Medicare group and the low-income younger group

^h^The coefficient of ‘Treatment∙Interruption∙Time’ indicates the change in slope difference in the likelihood of SNAP participation between the low-income older Medicare group and the younger group with low incomes

Further, the findings from our sensitivity analyses indicate that overall, similar patterns were shown, albeit with some minor differences (see Supplementary Tables S1 and S2). For instance, after incorporating a propensity score matching, the estimate of the parameter ‘Treatment∙Interruption∙Time’ as the main coefficient of interest shows that the difference in trend in the probability of SNAP participation after the interruption/intervention compared to pre-interruption/intervention between the low-income Medicare group and the younger with low incomes was estimated to be 2.6 PP (*P* < 0.001). Further, similar significant results were found for ‘Treatment∙Interruption’ (β =  − 0.123, *P* < 0.001), indicating that the level difference in the probability of SNAP enrollment after the intervention/interruption between the low-income older Medicare group and the younger with low incomes was estimated to be 12.3 PP. Furthermore, after employing a lower poverty threshold (≤ 130% of the FPL), we found that overall, a similar pattern was shown, even though the magnitude of effect was smaller. Notably, the estimate of the parameter ‘Treatment∙Interruption∙Time’ indicates that the difference in trend in the probability of SNAP participation after the interruption/intervention compared to pre-interruption/intervention between the low-income Medicare group and the younger with low incomes was estimated to be 2.3 PP (*P* < 0.001).

## Discussion

Based on a quasi-experimental design and using a national survey sample of Medicare beneficiaries, we found that the ACA, with its focus on the dual-eligible population, appeared to have a measurable and positive effect on SNAP participation among low-income older Medicare enrollees. Specifically, we observed a change in trend difference in the probability of SNAP enrollment from the pre- to post-intervention period to be 17.4 percentage points higher among low-income older Medicare enrollees compared to the low-income, SNAP-eligible younger individuals.

The increase in SNAP enrolment in the pre-intervention period (i.e. 2014) in almost every state in the US is largely attributable to increased demand and eligibility in the post-recession period, especially among younger working-class individuals, and due to a sluggish economic recovery [40, 41]. Between fiscal years 2007 and 2013, SNAP caseloads grew substantially, rising by almost 81%, and a significantly larger share of eligible people enrolled in the program [40]. Further, states’ continued efforts to reach and recruit more eligible households and individuals (especially low-income older adults) for the SNAP program contributed to the surge in participation post-recession. As computed and illustrated in our data and Fig. 2, and supported by prior evidence [40, 41], SNAP participation peaked in 2013 for low-income younger adults, however, for low-income Medicare enrollees, SNAP enrollment was sustained relatively at higher rates at least through 2016, when it finally plateaued.

Current estimates indicate that the overall number of SNAP participants fell by about 12% between 2013 and 2017, and decreased by almost 20% as of March 2019 [40, 42]. This downward trend is evident in Fig. 2. Another clear distinction as illustrated in this Figure is that during the pre-2014 period, rates of SNAP participation were increasing and were much higher among younger low-income adults, yet, this trend subsequently reversed mostly in the post-2014 era. Although SNAP enrollment rates showed a decline, they were declining at slower rates among low-income older Medicare beneficiaries as compared to low-income younger adults.

A number of factors likely contributed to the overall downward trends in the later years, including a slight increase in the total US population, and an improved economy [40]. Another factor that potentially contributed to a rise in SNAP participation, and had a reverse impact in 2016 and afterward, was the 2009 Recovery Act’s suspension of “SNAP’s three-month time limit on unemployed individuals aged 18–49 who are not raising minor children and did not have a serious disability,” to ease the negative impacts of the recession on the lower income individuals and families [40]. However, evidence indicates more than 500,000 adults aged 18–49 lost SNAP benefits in 2016 alone in states and areas that reinstated the three-month time limit that year, and by 2017, the rule was re-imposed in almost all of the US. This change further limited SNAP participation eligibility and uptake [40]. As a consequence, SNAP caseloads dropped by almost 1.5 million in 2016 [41].

Historically, eligible older adults have enrolled in SNAP at much lower levels compared to other age groups [43] despite SNAP’s potential benefit in reducing food insecurity and poverty [3]. It is plausible that dual-eligible individuals may be particularly hesitant to enroll in Medicaid, even when in need and would benefit from enrollment, because of perceived or realized stigma or other negative views of the program. For instance, health care for Medicaid recipients is often delayed or even refused [44]. Other evidence suggests that welfare stigma may adversely affect whether eligible individuals participate in government assistance programs such as SNAP [45]. It is possible that this stigma could be more widespread among poor older adults. If correct, this factor could discourage enrollment in means-tested welfare programs [46]. Despite these considerations, our study suggests that the ACA’s streamlining of the Medicaid application process for dual eligibles potentially boosted SNAP enrollment among low-income older Medicare beneficiaries. Nevertheless, many dually eligible individuals continue to remain unenrolled and further action may be warranted to address the remaining low SNAP participation among eligible seniors.

Some eligible older adults might believe that they do not need SNAP because they are receiving other benefits through government programs, including social security, which could generate an income effect [22]. Further, it is also likely that some older dual eligibles may be unaware of SNAP’s benefits or do not know how to apply for them, despite being in need [47]. Given that SNAP applications are available online in at least 44 states [20, 26], associated government workers, public health practitioners, other stakeholders, and outreach programs could help eligible seniors with SNAP applications by providing detailed assistance to apply, or at least provide relevant information and materials for that purpose. Evidence shows that most older adults who benefit from SNAP do live alone, and more than half are poor with little or no income [20]. Therefore, enrollment assistance and the average $1,248/year or $104/month of SNAP benefits could provide most of those who are eligible with necessary nutrition. Furthermore, offering additional assistance programs (e.g., Supplemental Security Income) or support mechanisms (e.g., transportation, social support, etc.) concurrently could enhance enrollment.

We found that among low-income White and Asian Medicare beneficiaries the likelihood of SNAP participation, after the policy change, increased by 13.7 PP and 40.8 PP, respectively; however, such results were not shown among older low-income Black Medicare beneficiaries. Furthermore, the probability of SNAP enrollment among non-Hispanic adults increased by 3.0 PP after the intervention; however, such a result was not found among Hispanics. Evidence suggests that African Americans, on average, have a higher level of mistrust of governmental programs, likely tied to a long history of social, systematic, and structural racism that may reduce participation in public welfare programs [48, 49]. African Americans and Hispanics, on average, belong to the lowest family/individual income category among all racial and ethnic groups, except for Native Americans, and are more likely to be food insecure [50]. Thus, reducing or eliminating barriers to participating in SNAP for them would provide significant health and societal benefits regarding racial and ethnic equality [3].

Our finding that the ACA increased SNAP participation among the lower-income older population suggests that similar future policies that link enrollment to multiple programs may lead to increased enrollment in SNAP. Policymakers should consider such policies, especially for those who are the most at risk of adverse health outcomes. Other studies also suggest that policies that are designed to reduce stigma for individuals in obtaining government assistance would also likely be of benefit. For instance, policies could be structured to provide universal meals, electronic or digital SNAP benefits, and unrestricted food choices among SNAP participants [51]. Finally, our finding that the ACA did not result in a differential increase in SNAP participation among older African and Hispanic American Medicare beneficiaries also points to the critical need to study and implement additional measures beyond those currently enacted under the ACA that are aimed at addressing disparities and ensuring equity in uptake for dual eligible African and Hispanic Americans.

The findings from the present study should be interpreted given several limitations. First, although we used a quasi-experimental design, the study approach may not fully explain the exact mechanism through which SNAP participation increased among low-income older Medicare enrollees. Second, there may be other related but unmeasured factors that were not adjusted for due to, for example, data limitations and the choice of our comparison group that comprised younger survey respondents. The study data lack information on such potentially relevant factors as household size, food insecurity and the receipts of social support. Similarly, potential biases could arise with the choice of similarly poor younger adults as control because they have different motivations and behaviors in the receipt of SNAP benefits when compared to their older counterparts. We address this issue by incorporating propensity score matching in our models to check the robustness and validity of our findings.

Third, although the study was based on a well-established model, the Program Logic Model, we cannot rule out the possibility of missing relevant, important factors influencing the results [28]. Fourth, due to limited data, we were unable to include State-level information in the analysis; however, this was not required in our comparative ITS design, as this study focused on the potential impact of the policy or intervention by the Dual Office, not Medicaid expansion per se, the latter of which varies across the States. Fifth, a small sample of Black people in the heterogeneity analysis could be the reason why the effects were not significant among Black people. The magnitudes of the coefficients are similar between Black and White people, but standard errors are larger for Black people (possibly due to a smaller sample size). Further, despite the fact that SNAP is a federal program with unified eligibility criteria, regional and state economic circumstances are usually taken into account to define qualification for SNAP. Moreover, state-to-state variations in administrative factors such as delays in processing caseloads and other administrative issues might influence SNAP participation [41]. One other critical factor leading to SNAP participation variations across states is that states may seek temporary waivers for the three-month benefits limit for unemployed non-disabled adults aged 18 to 49 who are not living with minor children for areas with high unemployment where qualifying jobs are scarce; these waivers could influence SNAP participation for the younger groups of low-income adults [22]. These and other limitations provide opportunities for future research.

## Conclusions

Using a national survey sample of Medicare beneficiaries and a quasi-experimental study design, we assessed the potential impact of the ACA in supporting the dual eligible program and its effect on SNAP participation among low-income older Medicare enrollees. Our findings are generalizable to all Medicare enrollees in this category in the US, during the studied period. Our ITS approach allowed for assessing the change in level and slope of SNAP participation related to policy change or intervention while adjusting for the overall trend in the outcome [52] and was relatively unaffected by conventional confounding factors [52, 53], and could theoretically adjust for potential concurrent events [53]. The findings strongly suggest that the ACA and its efforts to improve dual-eligible enrollment have potentially raised SNAP participation among low-income older Medicare enrollees in the US. Future studies could explore whether increased SNAP participation in older populations was also associated with improved health outcomes as has been shown with other groups of populations.

## Supplementary Information

**Additional file 1: Supplementary Table S1.** Comparative ITS analysis examining the ACA policy impact on SNAP participation among older Medicare enrollees with incomes ≤ 130% of the FPL. **Supplementary Table S2.** Comparative ITS analysis examining the ACA policy impact on SNAP participation among older Medicare enrollees with incomes ≤ 130% of the FPL using propensity score matching.

## Data Availability

The data analyzed for the current study were obtained from the IPUMS Medical Expenditure Panel Survey (MEPS) dataset[34], publicly available to access and download @ https://meps.ipums.org/meps/.

## Abbreviations

SNAP: Supplemental Nutrition Assistance Program
ACA: Affordable Care Act
US: United States
CMMI: Center for Medicare and Medicaid Innovation
MMCO: Medicare-Medicaid Coordination Office
ED: Emergency Department
MEPS: Medical Expenditure Panel Survey
FPL: Federal Poverty Level
CHIP: Children’s Health Insurance Program
ITS: Interrupted Time Series
PP: Percentage Points
