# Supplementary material for: Impact of the Affordable Care Act on participation in the Supplemental Nutrition Assistance Program among low-income older Medicare beneficiaries
Source: BMC Health Serv Res. 2023 May 19;23:509. doi: 10.1186/s12913-023-09557-7 (PMC10199570; doi:10.1186/s12913-023-09557-7)
Supplement: Supplementary file 1 — Additional file 1: Supplementary Table S1. Comparative ITS analysis examining the ACA policy impact on SNAP participation among older Medicare enrollees with incomes ≤ 130% of the FPL. Supplementary Table S2. Comparative ITS analysis examining the ACA policy impact on SNAP participation among older Medicare enrollees with incomes ≤ 130% of the FPL using propensity score matching. [file 12913_2023_9557_MOESM1_ESM.docx]

**Supplementary Table S1.** Comparative ITS analysis examining the ACA policy impact on SNAP participation among older Medicare enrollees with incomes ≤ 130% of the FPL.

|  |  | **SNAP participation** | | | |
| --- | --- | --- | --- | --- | --- |
| Parameter | Interpretation | β | *P* | SE ^a^ | t |
| Time ^b^ | Control Pre–Trend | 0.031 | <.001 | .003 | 9.59 |
| Interruption ^c^ | Control Post–Level Change | 0.021 | .04 | .010 | 2.02 |
| Time∙Interruption ^d^ | Control Post–Trend Change | −0.049 | <.001 | .004 | −11.82 |
| Treatment ^e^ | Treatment/Control Pre–Level Difference | −0.112 | <.001 | .013 | −8.24 |
| Treatment∙Time ^f^ | Treatment/Control Pre–Trend Difference | −0.007 | .15 | .005 | −1.40 |
| Treatment∙Interruption ^g^ | Treatment/Control Post–Level Difference | −0.002 | .89 | .017 | −0.12 |
| Treatment∙Interruption∙Time ^h^ | Treatment/Control Change in Slope Difference Pre– to Post– | 0.023 | <.001 | .006 | 3.49 |
| *Abbreviations*. ITS; interrupted time series; ACA, Affordable Care Act; SNAP, Supplemental Nutrition Assistance Program; β, unstandardized beta coefficient; *P*, p-value; SE, standard error; t, t-statistic; FPL, Federal Poverty Level.  *Notes.* Control group included younger individuals aged 27 to 64 years with incomes ≤ 130% of the FPL. | | | | | |
| ^a^ Newey-West autocorrelation adjusted standard error. | | |  |  |  |
| ^b^ The coefficient of ‘Time’ indicates the trend (or slope) in the probability of SNAP participation in the low-income younger group before interruption/intervention. | | | | | |
| ^c^ The coefficient of ‘Interruption’ shows the level change in the likelihood of SNAP participation right after the interruption/intervention in the low-income younger group. | | | | | |
| ^d^ The coefficient of ‘Time∙Interruption’ indicates the trend (or slope) change in the probability of SNAP participation after the interruption in the low-income younger group. | | | | | |
| ^e^ The coefficient of ‘Treatment’ shows the level difference in the probability of SNAP enrollment before the intervention between the low-income older Medicare group and the low-income younger group. | | | | | |
| ^f^ The coefficient of ‘Treatment∙Time’ indicates the trend (or slope) difference in the probability of SNAP participation before intervention between the low-income older Medicare group and the low-income younger group. | | | | | |
| ^g^ The coefficient of ‘Treatment∙Interruption’ shows the level difference in the probability of SNAP enrollment between the low-income older Medicare group and the low-income younger group. | | | | | |
| ^h^ The coefficient of ‘Treatment∙Interruption∙Time’ indicates the change in slope difference in the likelihood of SNAP participation between the low-income older Medicare group and the younger group with low incomes. | | | | | |

**Supplementary Table S2.** Comparative ITS analysis examining the ACA policy impact on SNAP participation among older Medicare enrollees with incomes ≤ 130% of the FPL using propensity score matching

|  |  | **SNAP participation** | | | |
| --- | --- | --- | --- | --- | --- |
| Parameter | Interpretation | β | *P* | SE ^a^ | t |
| Time ^b^ | Control Pre–Trend | 0.036 | <.001 | .010 | 3.50 |
| Interruption ^c^ | Control Post–Level Change | 0.142 | <.001 | .022 | 6.37 |
| Time∙Interruption ^d^ | Control Post–Trend Change | −0.053 | <.001 | .010 | -4.95 |
| Treatment ^e^ | Treatment/Control Pre–Level Difference | 0.039 | .14 | .027 | 1.45 |
| Treatment∙Time ^f^ | Treatment/Control Pre–Trend Difference | −0.013 | .22 | .010 | -1.20 |
| Treatment∙Interruption ^g^ | Treatment/Control Post–Level Difference | −0.123 | <.001 | .023 | -5.22 |
| Treatment∙Interruption∙Time ^h^ | Treatment/Control Change in Slope Difference Pre– to Post– | 0.026 | .01 | .011 | 2.42 |
| *Abbreviations*. ITS; interrupted time series; ACA, Affordable Care Act; SNAP, Supplemental Nutrition Assistance Program; β, unstandardized beta coefficient; *P*, p-value; SE, standard error; t, t-statistic; FPL, Federal Poverty Level.  *Notes*. 1) Control group included younger individuals aged 27 to 64 years with incomes ≤ 130% of the FPL. 2) ITS analyses were based on the data that matched each older Medicare enrollee to the younger based on propensity score, calculated based on the covariates (age, sex, race, education, marital status, and income). | | | | | |
| ^a^ Cluster robust standard error. | | |  |  |  |
| ^b^ The coefficient of ‘Time’ indicates the trend (or slope) in the probability of SNAP participation in the low-income younger group before interruption/intervention. | | | | | |
| ^c^ The coefficient of ‘Interruption’ shows the level change in the likelihood of SNAP participation right after the interruption/intervention in the low-income younger group. | | | | | |
| ^d^ The coefficient of ‘Time∙Interruption’ indicates the trend (or slope) change in the probability of SNAP participation after the interruption in the low-income younger group. | | | | | |
| ^e^ The coefficient of ‘Treatment’ shows the level difference in the probability of SNAP enrollment before the intervention between the low-income older Medicare group and the low-income younger group. | | | | | |
| ^f^ The coefficient of ‘Treatment∙Time’ indicates the trend (or slope) difference in the probability of SNAP participation before intervention between the low-income older Medicare group and the low-income younger group. | | | | | |
| ^g^ The coefficient of ‘Treatment∙Interruption’ shows the level difference in the probability of SNAP enrollment between the low-income older Medicare group and the low-income younger group. | | | | | |
| ^h^ The coefficient of ‘Treatment∙Interruption∙Time’ indicates the change in slope difference in the likelihood of SNAP participation between the low-income older Medicare group and the younger group with low incomes. | | | | | |
